# Effects of Nano-Titanium Dioxide on Freshwater Algal Population Dynamics

**DOI:** 10.1371/journal.pone.0047130

**Published:** 2012-10-10

**Authors:** Konrad J. Kulacki, Bradley J. Cardinale

**Affiliations:** 1 Department of Ecology, Evolution, and Marine Biology, University of California Santa Barbara, Santa Barbara, California, United States of America; 2 School of Natural Resources and Environment, University of Michigan, Ann Arbor, Michigan, United States of America; RMIT University, Australia

## Abstract

To make predictions about the possible effects of nanomaterials across environments and taxa, toxicity testing must incorporate not only a variety of organisms and endpoints, but also an understanding of the mechanisms that underlie nanoparticle toxicity. Here, we report the results of a laboratory experiment in which we examined how titanium dioxide nanoparticles impact the population dynamics and production of biomass across a range of freshwater algae. We exposed 10 of the most common species of North American freshwater pelagic algae (phytoplankton) to five increasing concentrations of n-TiO_2_ (ranging from controls to 300 mg n-TiO_2_ L^−1^). We then examined the effects of n-TiO_2_ on the population growth rates and biomass production of each algal species over a period of 25 days. On average, increasing concentrations of n-TiO_2_ had no significant effects on algal growth rates (*p* = 0.376), even though there was considerable species-specific variation in responses. In contrast, exposure to n-TiO_2_ tended to increase maximum biomass achieved by species in culture (*p* = 0.06). Results suggest that titanium dioxide nanoparticles could influence certain aspects of population growth of freshwater phytoplankton, though effects are unlikely at environmentally relevant concentrations.

## Introduction

Engineered nanomaterials are a rapidly expanding group of materials in which industrial, academic, and consumer interest has been steadily growing [1]. Loosely defined as manufactured materials that are smaller than 100 nanometers in at least one dimension [2], nanomaterials have been of scientific interest for several decades, but are now being used in a wide range of commercial applications. Titanium dioxide (n-TiO_2_) is one of the most widely produced nanoparticles [3], and because of its photoactivity, whitening ability, and transparency in the nanoparticulate form, it is widely used in paints, sunscreens, cosmetics, and solar technologies [1], [4]. Like most nanomaterials, the development and use of n-TiO_2_ epitomizes a growing problem – while an ever increasing number of new technologies and products are taking advantage of the unique properties of n-TiO_2_, rarely have the potential hazards of these materials been effectively assessed. Given the predicted trends of increasing production of n-TiO_2_, there is an increasing likelihood of its release into the environment via industrial waste, wastewater effluent, personal health care products (PHP’s), and the weathering of painted surfaces [3].

As n-TiO_2_ is released into the environment, one of the primary concerns is that it may come into contact with, and potentially impact, aquatic organisms. Summaries suggest that primary producers, such as freshwater algae that form the base of many food-webs, may be sensitive to metal oxides like n-TiO_2_, and could be impacted more than other types of organisms [5]. While this may be true, it is worth noting that studies have shown considerable variability in the response of pelagic algae to n-TiO_2_. Several studies performed with the common laboratory model organism *Pseudokirchneriella subcapitata* have reported toxic effects of n-TiO_2_, but EC-50 values have ranged widely (reviewed in [6]). In contrast, n-TiO_2_ has been shown by others to have no effect on *P. subcapitata* photosynthetic activity after short-term (4.5 h) exposure at concentrations up to 100 mg n-TiO_2_ L^−1^ [7], and may even stimulate *P. subcapitata* growth rates at low concentrations (0–10 mg L^−1^) [8]. While experimental duration and particle characteristics varied across these tests, the contrast among results to date suggest that we do not yet have a firm idea of how n-TiO_2_ generally impacts pelagic algae, and that a broader range of testing will be necessary to resolve these inconsistencies.

Testing of the effects of n-TiO_2_ on freshwater primary producers to date has focused on a limited set of taxa and endpoints (e.g., growth or photosynthesis). Aside from the several studies using *P. subcapitata*, only five other taxa have been used in toxicological testing of n-TiO_2_: four green algae (Chlorophyta: *Desmodesmus subspicatus*, *Chlamydomonas reinhardtii*, *Chlorella* spp., and *Scenedesmus* spp) and one cyanobacteria (*Anabaena variabilis*) [9]–[12]. Based on a 2007 survey done by the U.S. EPA, *Anabaena*, *Scenedesmus*, *Chlorella*, and *Chlamydomonas* were the 4^th^, 5^th^, 21^st^, and 36^th^ most common genera seen in 1154 lakes sampled across the United States [13]. *Desmodesmus* and *Pseudokirchneriella* did not appear in any samples. Given that more than 770 genera of freshwater phytoplankton are known to exist in North America alone [14], it seems critical that we expand our testing to include a broader range of taxa that includes multiple growth forms, taxonomic groups, and natural frequencies. In addition, most testing to date has primarily focused on acute effects (<96 h) on algal growth rates [8], [9], [15]–[17]. This response variable has rather limited ability to predict the response of algal growth rates and carrying capacities to anthropogenic chemicals [18]. Changes in both the carrying capacity of species and their ability to be preyed upon by higher trophic levels have major implications for eutrophication and the potential for harmful algal blooms [19]–[21], as well as the trophic transfer of materials to higher consumers in a food chain.

The objectives of our study were to determine how n-TiO_2_ affects the growth rates and production of biomass by a broad range of freshwater phytoplankton. We exposed 10 different species of diatoms, green algae, and cyanobacteria, to five increasing concentrations of n-TiO_2_ (0–300 mg L^−1^) in 1-L glass bottles. We then measured algal growth rates and the maximum biomass attained (an index of carrying capacity) over a 25-day period of growth. This work represents the broadest taxonomic investigation of the effects of nanoparticles on freshwater phytoplankton to date.

## Methods

### Experimental Units

The experimental units used for the study were 1-L clear, borosilicate Wheaton roller bottles containing soil extract growth media [22]. The growth media was prepared by adding 0.09-L of dry greenhouse potting soil and 0.05-g magnesium carbonate to 1-L of ultrapure water. The mixture was allowed to steep for 48 h, after which it was passed through a 10-um filter to remove particulates. To each roller bottle, we added 250-ml of media, and each bottle was autoclave sterilized for 1 h. We used soil extract media for this experiment for two reasons. First, soil extract is one of the few media that allow for natural growth of algae that span vastly different taxonomic groups, such as the variety of species used in this experiment. By comparison, most of the synthetic culture media that are commonly used in ecotoxicology studies (e.g., COMBO, Chu, Bold’s Basal, etc.) are specifically tailored to the growth of particular groups of algae and, as such, are usually insufficient for culturing divergent taxonomic groups in the same experiment. Second, compared to synthetic culture media, soil extract media more closely resembles several important physical and chemical properties of natural surface waters, including the presence of natural organic matter, and, as such, exhibits characteristics that are likely to influence the behavior of, and exposure to, n-TiO_2_ in natural systems. In a companion study to this project [23], we published a detailed and extensive characterization of the physical and chemical properties of soil extract media (labeled mesocosm freshwater throughout reference [23]) to examine how the properties of this liquid media influence the aggregation and sedimentation rates of n-TiO_2_ particles that control exposure. Based on results of this study, we have provided information in Table S1 that details the chemical composition of the soil extract media, and show in Figures S1 and S2 the average particle size and sedimentation rates over time of n-TiO_2_ in this media.

### Test Species and Culture Conditions

The algal species used in this experiment are listed in Table 1. All taxa were either obtained from culture providers or isolated from locally collected samples (source is indicated in Table 1). No specific permits were required for the locally collected samples; all samples were obtained from publicly accessible waters and none of these taxa are endangered or protected. Cultures in the laboratory were maintained in an incubator under a 12 h:12 h light/dark cycle at 15°C, and were grown on petri dishes made with soil extract media and 2% agar. Approximately 4 weeks prior to use, cultures were transferred to liquid soil extract media for growth to batch culture densities. All cultures were confirmed to be free of contamination by other algal species prior to use; however, the cultures were not established to be axenic, and bacteria were almost certainly present. All 1-L roller bottles were inoculated with 0.024±0.008 µg chlorophyll-*a* from the appropriate algal batch culture immediately prior to the start of the experiment.

**Table 1:** Algal strains used in this experiment, along with their frequency in North American lakes.

Species name	Taxonomic group	Common form	Frequency (%)	Rank Freq.	Rank Abundance
*Anabaena spp.* 1	Cyanobacteria	Filaments	55.9	4	13
*Navicula subminuscula* 1	Bacillariophyta	Individual cells	35.4	16	168
*Nitzschia pusilla* 2	Bacillariophyta	Individual cells	31.1	22	116
*Oscillatoria spp.* 1	Cyanobacteria	Filaments	33.7	19	24
*Planothidium lanceolatum* 2	Bacillariophyta	Individual cells	N/A		
*Scenedesmus quadricauda* 3	Chlorophyta	Colonies	52.9	5	51
*Selenastrum minutum* 3	Chlorophyta	Individual cells	16.5	48	147
*Spirogyra communis* 3	Charophyta	Filaments	0.34	183	184
*Stigeoclonium tenue* 3	Chlorophyta	Individual cells	0.86	139	216
*Tabularia fasciculata* 2	Bacillariophyta	Individual cells	5.46	83	145

1 Obtained from the Carolina Biological Supply Company (North Carolina, USA).

2 Isolated in the laboratory from local environmental samples.

3 Obtained from the University of Texas Culture Collection (UTEX; Texas, USA).

N/A: Genus not observed in the 2007 survey.

Frequency (%): percentage of lakes in which this genus was present, based on a 2007 survey completed by the U.S. EPA (n = 1154 lakes) [13].

Rank Freq: of the 262 total genera found, ranking based on how frequently the particular genus was observed.

Rank Abundance: ranking based on the average abundance of genera in all lakes where present.

**Table 2:** Slopes, intercepts, R^2^, and *p* values for each regression line of GR_max_ or B_max_ versus n-TiO_2_ exposure concentration.

	GR_max_ versus [n-TiO_2_]				B_max_ versus [n-TiO_2_]			
	Slope	Intercept	R^2^	*p*	Slope	Intercept	R^2^	*p*
*Anabaena spp.*	−0.00021	0.340	0.114	0.978	0.065	853.4	0.000	0.579
*Navicula subminuscula*	−0.00061	0.385	0.663	0.117	4.529	596.8	0.614	0.093
*Nitzschia pusilla*	−0.00012	0.753	0.107	0.342	1.419	1545.8	0.297	0.590
*Oscillatoria spp.*	−0.00013	0.150	0.045	0.646	−0.308	234.4	0.079	0.732
*Planothidium lanceolatum*	0.00104	0.602	0.307	0.020	4.408	770.2	0.873	0.332
*Scenedesmus quadricauda*	0.00062	0.319	0.858	0.052	8.592	1128.0	0.765	0.024
*Selenastrum minutum*	0.00027	0.453	0.541	0.142	4.864	1145.0	0.566	0.156
*Spirogyra communis*	−0.00084	0.738	0.898	0.306	−3.794	2369.5	0.336	0.014
*Stigeoclonium tenue*	0.00296	0.224	0.807	0.131	18.259	1948.6	0.587	0.038
*Tabularia fasciculata*	0.00020	0.688	0.030	0.269	3.009	1339.8	0.379	0.782

Example plots of *Scenedesmus quadricauda* and *Anabaena spp.* are given in Figure S4.

### Experimental Set-up

Our study was designed to test effects of n-TiO_2_ on algae using a regression-based approach in which each algal species was exposed to five increasing concentrations of the nanoparticles (0 control, 50, 100, 200, and 300 mg n-TiO_2_ L^−1^). Each particle concentration was replicated only 1x per species; thus, there were 10 species×5 particle concentrations, for a total of 50 roller bottles. We specifically chose to use a regression-based approach (as opposed to a replicated ANOVA-type exposure) because having fewer replicates and more treatments spanning an x-axis of initial concentration allows one to generate quantitative estimates of covariance [24]. A limitation of this technique is that less replication can lead to low statistical power and difficulty detecting effects, however in our case we were generally able to detect significant responses (see Results). These particle concentrations were chosen to mimic those used by Hartmann et al. [8], who saw inhibitory effects of the same particles at concentrations ranging from 10–250 mg n-TiO_2_ L^−1^.

The n-TiO_2_ used in this study was purchased from Evonik Degussa Corp (CAS# 13463-67-7), and was comprised of 82% anatase/18% rutile TiO_2_ (98% pure). The initial particle size was 27±4 nanometers, with particles having a specific surface area of 50±15 m^2^ g^−1^. The n-TiO_2_ stock solution was prepared in Milli-Q water and sonicated for 30 minutes in a bath sonicator prior to addition to roller bottles. As n-TiO_2_ is not soluble in water [8], [25], [26], we did not test the toxicity of dissolved Ti ions. After adding Ti to the roller bottles in the appropriate concentrations, bottles were placed on a roller rack (BellCo Digital Top Drive Roller apparatus) where they were rotated constantly at 6.5 rpm for the duration of the experiment. This roller rack was illuminated by twenty-two Phillips 32-watt coolwhite T8 fluorescent lights set to a 16 h:8 h light:dark photoperiod. Using a UV AB meter (UV513AB, General Tools, New York, NY), ultraviolet light output from these bulbs was approximately 113 µW cm^−2^. The entire apparatus was located in a walk-in environmental chamber that was set to a constant 18°C.

### Sampling

Sampling of the algae was performed every 2–4 days (9 times total) over the course of 25 days. This duration was sufficient to achieve 3–4 doublings of biomass of most species, and was sufficient time for most of the experimental bottles to achieve a steady-state biomass (Figure S3). On each sampling day, test bottles were removed from the roller rack, homogenized for ca. 20 seconds using a handheld mixer, and 3-mL of unfiltered water sample was removed and analyzed for fluorescence on a handheld fluorometer (Aquafluor, Turner Designs). Each sample was analyzed in triplicate to ensure adequate characterization of the experimental unit. Values were corrected for background fluorescence of n-TiO_2_ based on measurements taken in algal-free control bottles.

### Data Analysis

Our experimental units were treated as ‘batch cultures’ (as opposed to chemostats), whereby growth media was not replenished over the course of the experiment. The typical time-series of algal growth in batch cultures involves logistic growth followed by a decline in algal biomass once nutrients are depleted (see Fig. S3 for growth curves of algae used in this experiment). The decline phase makes fitting of traditional growth equations (e.g. Gompertz functions) to the time-series of batch cultures problematic at best, impossible at worst. Because of this, we decided to summarize the time-series of each batch culture using two easily interpretable metrics that give the maximum potential growth rate of a species *GR_max_* (a proxy for *r*, the instantaneous per biomass rate of increase) and the maximum biomass *B_max_* achieved in culture. The maximum specific growth rate (day^−1^) was calculated as the largest proportional increase in biomass measured between any two consecutive sampling days in a given culture. Note that because species grew at different rates, and because growth may have been influenced by Ti exposure, the sampling dates used to calculate *GR_max_* were specific to each culture bottle. *B_max_* was taken to be the highest biomass achieved by an algal species in a given culture bottle on any date. This measure should not necessarily be equated with a carrying capacity *K*, since overshoot can produce transient values of biomass that cannot be sustained in culture. Nevertheless, *B_max_* is an index of the maximum potential efficiency for algae to use limited resources and convert those into new tissue.

We regressed *GR_max_* and *B_max_* against exposure concentration of n-TiO_2_, using a one sided t-test to assess whether there was significant impacts of Ti on the growth trajectories of each algal species.

## Results

**Figure 1:**
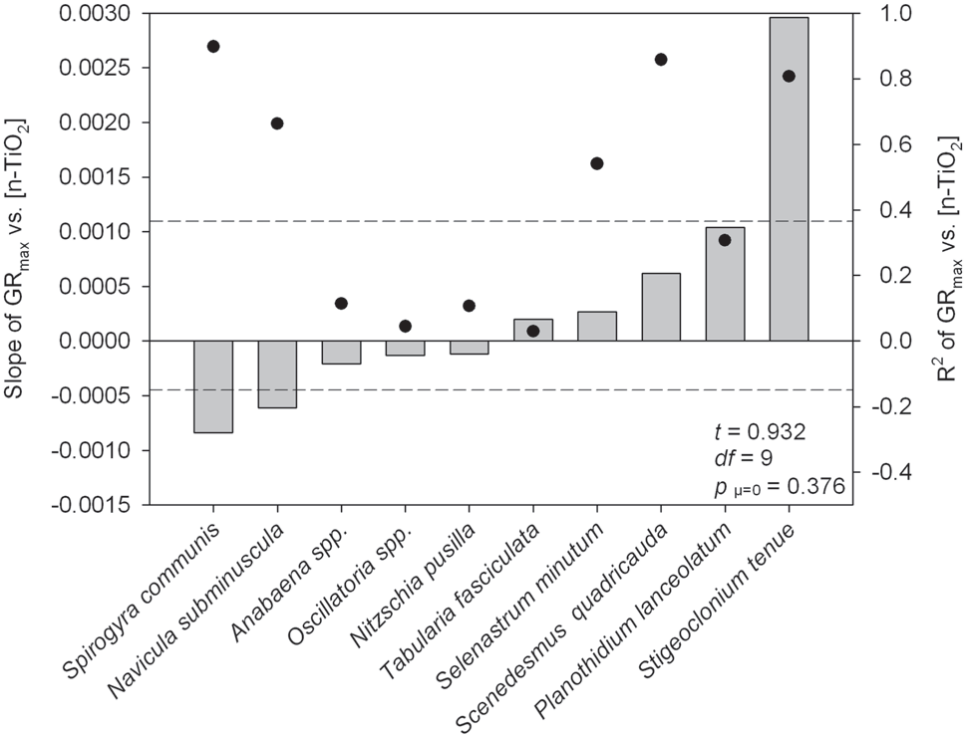
Effects of increasing concentrations of n-TiO_2_ on maximum algal growth rates. Slopes of maximum algal growth rate (GR_max_) versus exposure concentration of n-TiO_2_ ([n-TiO_2_], in mg L^−1^). Dots represent the R^2^ of the trendline represented by each bar. Statistics shown relate to a 1-sample t-test, comparing the average of the slopes to a null H_o = _0. Dotted lines represent the 95% confidence intervals around the mean of all slopes. Positive (or negative) bars indicate that increasing exposure concentrations of n-TiO_2_ had increasingly positive (or negative) effects on GR_max_.

When averaged across all ten species considered in this study, n-TiO_2_ had no significant influence on the maximum potential growth rate of freshwater algae (*p* = 0.376; Fig. 1, dashed lines show 95% confidence interval for *GR_max_*). This is not to say that n-TiO_2_ had no impacts on the growth of individual algal species. To the contrary, Figure 1 shows considerable variation in species-specific responses to n-TiO_2_ - responses that span from strong inhibition of maximum potential growth rates (e.g., *Spirogyra communis*) to strong stimulation of maximum potential growth rate (e.g., *Stigeoclonium tenue*). The slopes of the individual regression lines for *S. communis, S. quadricauda*, and *S. tenue* were all significantly different from zero (*p*<0.05); however, when averaged across all of the taxa considered, the 95% confidence interval for the slopes relating *GR_max_* to n-TiO_2_ was not (Fig. 1 dashed lines). Slope, intercept, R^2^, and *p*-values for all species can be found in Table 2. It is worth noting that the amount of variation in *GR_max_* that was explained by variation in n-TiO_2_ was low for five of the focal species (R^2^<0.40, Figure 1). The low explanatory power could represent a true lack of response to n-TiO_2_, or alternatively, it could suggest weak and/or idiosynchratic responses that require more data to detect a response.

**Figure 2:**
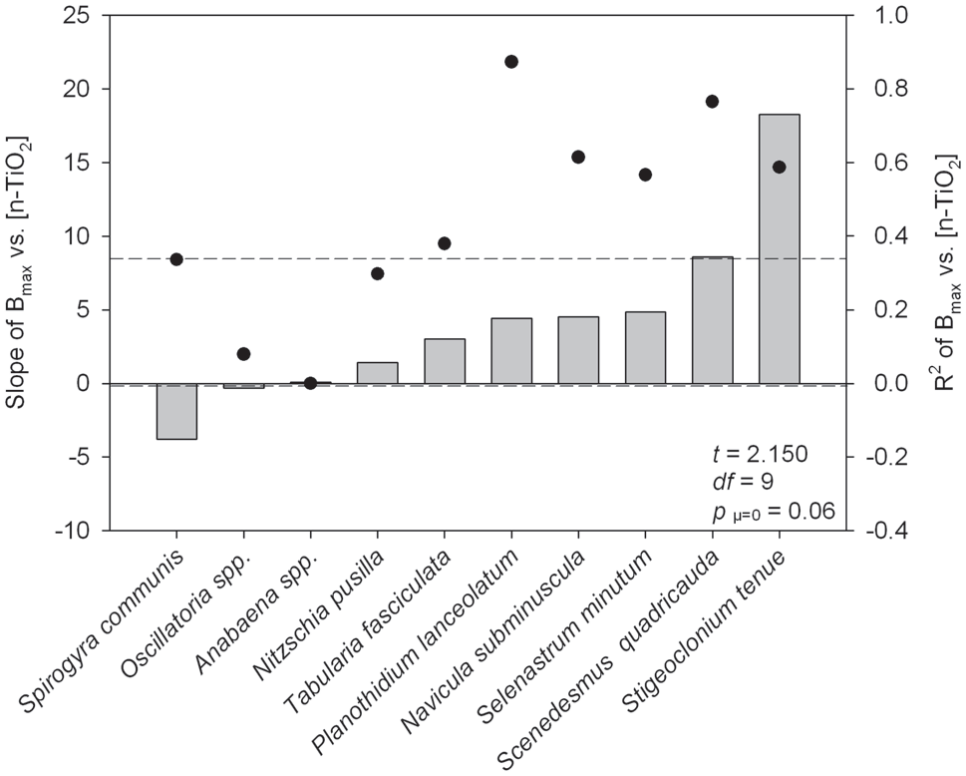
Effects of increasing concentrations of n-TiO_2_ on maximum algal biomass. Slopes of maximum algal biomass (as measured by fluorescence; B_max_) versus exposure concentration of n-TiO_2_ ([n-TiO_2_], in mg L^−1^). Dots represent the R^2^ of the trendline represented by each bar. Statistics shown relate to a 1-sample t-test, comparing the average of the slopes to a H_o = _0. Dotted lines represent the 95% confidence intervals around the mean of all slopes. Positive (or negative) bars indicate that increasing exposure concentrations of n-TiO_2_ had increasingly positive (or negative) effects on B_max_.

Increasing concentrations of n-TiO_2_ also appeared to impact maximum biomass algae achieved in culture (Fig. 2). A t-test to differentiate the mean slope of the regression lines from zero had a p-value of 0.06, which we interpret as evidence that n-TiO_2_ generally increased maximum biomass. Seven of the ten species exposed showed a positive relationship between maximum biomass and n-TiO_2_ exposure concentration (Fig. 2). The slope for *Planothidium lanceolatum* was different from zero (*p*<0.05). The amount of variation in *B_max_* that was explained by variation in n-TiO_2_ was again low for five of our ten focal species, with four of these species also showing a weak relationship between *GR_max_* and n-TiO_2_ concentration: *Anabaena spp.*, *Oscillatoria spp.*, *Nitzschia pusilla*, and *Tabularia fasciculata*. These species may simply be unaffected by n-TiO_2_, though additional testing is necessary to confirm.

## Discussion

In the current study, we found that n-TiO_2_ has little effect on algal growth rates. This was driven by the fact that of our ten species, some responded negatively to increasing concentrations of n-TiO_2_, while others responded positively. Our results contrast with many other studies to date, which have typically shown that n-TiO_2_ strongly decreases algal growth rates [8], [10], [15]–[17], including two taxa similar to those used in our study (*Anabaena variabilis* [12] and *Scenedesmus spp.* [11]). While the latter study used particles similar to ours (25-nm reported particle size, predominantly anatase crystal structure [11]), the former study used 10-nm nTiO_2_ particles [12]. Particle size and structure have been shown to affect toxicity [8], [27], and the different particles used may explain some of the differences seen in responses of the algae to n-TiO_2_ exposure.

Perhaps more importantly though, all of these previous studies have used 1) a limited number of taxa, 2) short (≤96 h) experimental durations, and 3) test media that did not mimic natural waters. Our study is the first to examine such a broad range of freshwater algal taxa under identical experimental conditions using a “natural” media, to extend the test duration past exponential growth phase, and the first to document a range of negative to positive effects of n-TiO_2_ on algal growth across a broad range of taxa. While other studies used a select few common taxa, our work shows that, given a broader range of taxa used in toxicity testing, we may see a broader range of responses to nanoparticle exposure.

When analyzed as a group, n-TiO_2_ tends to have positive effects on maximum algal biomass. Based on results of this study, it is unclear how n-TiO_2_ might increase maximum biomass. But here we propose three, non-mutually exclusive mechanisms that might help guide further research. First, n-TiO_2_ could reduce competition with bacteria for limiting nutrients. Nanoparticles, including n-TiO_2_, have been shown to damage the cell membranes of prokaryotes [28], [29], and if the effect on bacteria is greater than any negative effect on algae, then algae could benefit from reduced competition for nutrients. To our knowledge, this hypothesis has not been directly tested; however, a recent review suggested that algae are 10–100 times more sensitive than bacteria to TiO_2_ nanoparticles [5].

Second, n-TiO_2_ has been shown to photoactivate in the presence of UV radiation, which leads to the generation of reactive oxygen species (ROS) [9]. While ROS are typically thought of as having negative effects on algae and other organisms [9], [30], these same oxygen radicals have the potential to break down natural organic matter and release nutrients that might stimulate the growth of algae and bacteria [31], [32]. While the lights used in our experiment were emitting 113 µW cm^−2^ of light in the UV range, it is unclear whether this was enough lead to breakdown of NOM. Future studies will more explicitly address this possibility.

A third possible mechanism for the increases in B_max_ seen in this experiment could be that the algae were simply increasing cellular chlorophyll content. n-TiO_2_ has been shown to coat algal cells [8], which could lead to shading at the individual cell level. Algae have been shown to increase cellular chlorophyll in response to low light conditions [33], [34], and thus the increases seen in B_max_ may represent increases in cellular chlorophyll content. Fluorescence is a commonly measured endpoint in algal toxicological testing [35], and thus future studies should make use of other endpoints to corroborate observed effects.

### Limitations and Caveats

In most natural and test media, engineered nanoparticles are unlikely to remain in nano form. Particle aggregation will therefore undoubtedly influence the concentrations of nanoparticles to which organisms are exposed. In standard laboratory tests, the formation of nanoparticle aggregates is common [8], [15], and may in fact approximate environmentally relevant exposures more so than artificially keeping particles in nano form (e.g., using dispersants) [36]. In our test media, n-TiO_2_ has been shown to form aggregates approximately 300-nm in size that are stable over a period of at least seven hours [21]. In addition, over that time only a small proportion (<5%) of the initial nanoparticle exposure settled out of solution. While particle aggregation and sedimentation rates are expected to be much higher in marine environments [21], these results suggest that in our test media, chosen in part for its semblance to natural surface waters, exposure concentrations should remain high over the duration of the test.

The exposure concentrations used in our study ranged from 0 to 300 mg n-TiO_2_ L^−1^, and are comparable to those of previous studies [8], [14]. However, environmentally relevant concentrations are expected to be in the range of 0.7–600 µg n-TiO_2_ L^−1^ [37], [38]. When these expected concentrations are combined with the magnitude of effects seen in our study, it suggests that the effects of n-TiO_2_ on freshwater phytoplankton may in fact be negligible, though numerous other environmental variables (e.g., presence of other chemical stressors, greater levels of UV light) could interact and cause unforeseen effects.

### Implications

Our study is the first to look at the effects of n-TiO_2_ on a wide range of algal taxa. We saw no effects of n-TiO_2_ on algal growth rates and scattered positive effects on maximum algal culture biomass. The generality of these results indicate that the same mechanisms may be responsible for the effects of n-TiO_2_ on most freshwater phytoplankton, and that comparing impacts across species for risk assessments should be possible. While our study has shown some possible impacts of n-TiO_2_ on freshwater phytoplankton, more work is needed to investigate underlying mechanisms, especially as it relates to the disparity seen across studies to date. Future studies should aim to go beyond standard toxicity testing, and include a broader range of species, both acute and chronic endpoints, and more realistic environmental conditions. Only by pairing comprehensive studies on the effects of engineered nanoparticles on freshwater organisms with realistic exposure scenarios that incorporate the range of conditions seen in nature will we be able to fully assess their risks to natural environments.

## Supporting Information

Figure S1 **n-TiO2 aggregation in soil-water media over time.** Aggregate sizes of n-TiO_2_ over time, measured by dynamic light scattering, at three initial particle concentrations (10, 50, and 100 mg L^−1^). Reprinted with permission from Keller et al., Stability and Aggregation of Metal Oxide Nanoparticles in Natural Aqueous Matrices, *Environmental Science and Technology*. Copyright 2010, American Chemical Society.
(TIF)

Figure S2 **Sedimentation rates of n-TiO_2_ in soil-water media over time.** Sedimentation rates, shown at four initial concentrations (10, 50, 100, and 200 mg L^−1^). Reprinted with permission from Keller et al., Stability and Aggregation of Metal Oxide Nanoparticles in Natural Aqueous Matrices, *Environmental Science and Technology*. Copyright 2010, American Chemical Society.
(TIF)

Figure S3 **Growth trajectories for each of the ten species used in this experiment.** A: *Anabaena spp.*; B: *Navicula subminuscula*; C: *Scenedesmus quadricauda*; D: *Nitzschia pusilla*; E: *Oscillatoria spp.*; F: *Selanastrum minutum*; G: *Spirogyra communis*; H: *Planothidium lanceolatum*; I: *Tabularia fasciculate*; J: *Stigeoclonium tenue*.
(TIF)

Figure S4 **Example plots showing how data for** **Table 2** **were generated.** The effects of n-TiO_2_ exposure concentration on the maximum growth rate (GR_max_, top panel) and maximum biomass as measured by fluorescence (B_max_, bottom panel) of *Scenedesmus quadricauda* (filled dots) and *Anabaena spp.* (open dots). From the regression lines plotted here, the slopes, intercepts, and R^2^ values for these, and all other, algal species are given in Table 2 in the main text, and the slopes and R^2^ values are plotted in Figures 1 and 2.
(TIF)

Table S1 **Characteristics of the soil extract media used in this study, as measured in a companion study.** Reprinted with permission from Keller et al., Stability and Aggregation of Metal Oxide Nanoparticles in Natural Aqueous Matrices, *Environmental Science and Technology*. Copyright 2010, American Chemical Society.
(DOCX)
