# Supplementary figures and images for: Effects of Nano-Titanium Dioxide on Freshwater Algal Population Dynamics
Source: PLoS One. 2012 Oct 10;7(10):e47130. doi: 10.1371/journal.pone.0047130 (PMC3468504; doi:10.1371/journal.pone.0047130)

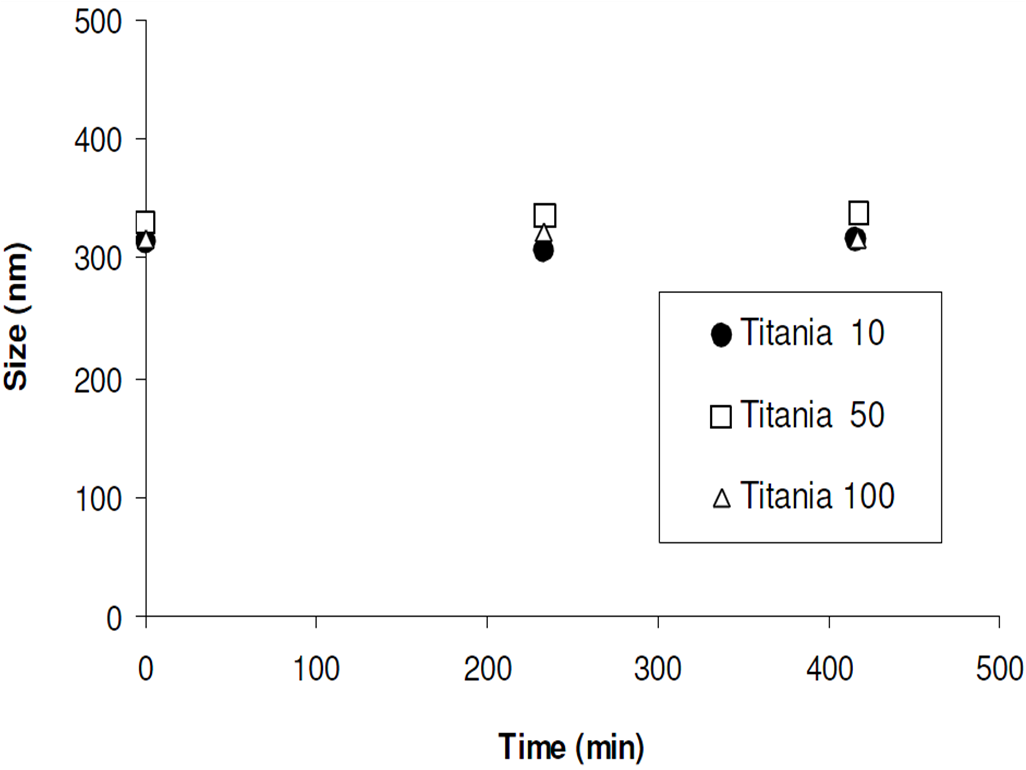

Supplement: Figure S1 — n-TiO2 aggregation in soil-water media over time. Aggregate sizes of n-TiO2 over time, measured by dynamic light scattering, at three initial particle concentrations (10, 50, and 100 mg L−1). Reprinted with permission from Keller et al., Stability and Aggregation of Metal Oxide Nanoparticles in Natural Aqueous Matrices, Environmental Science and Technology. Copyright 2010, American Chemical Society. (TIF) [file pone.0047130.s001.tif]

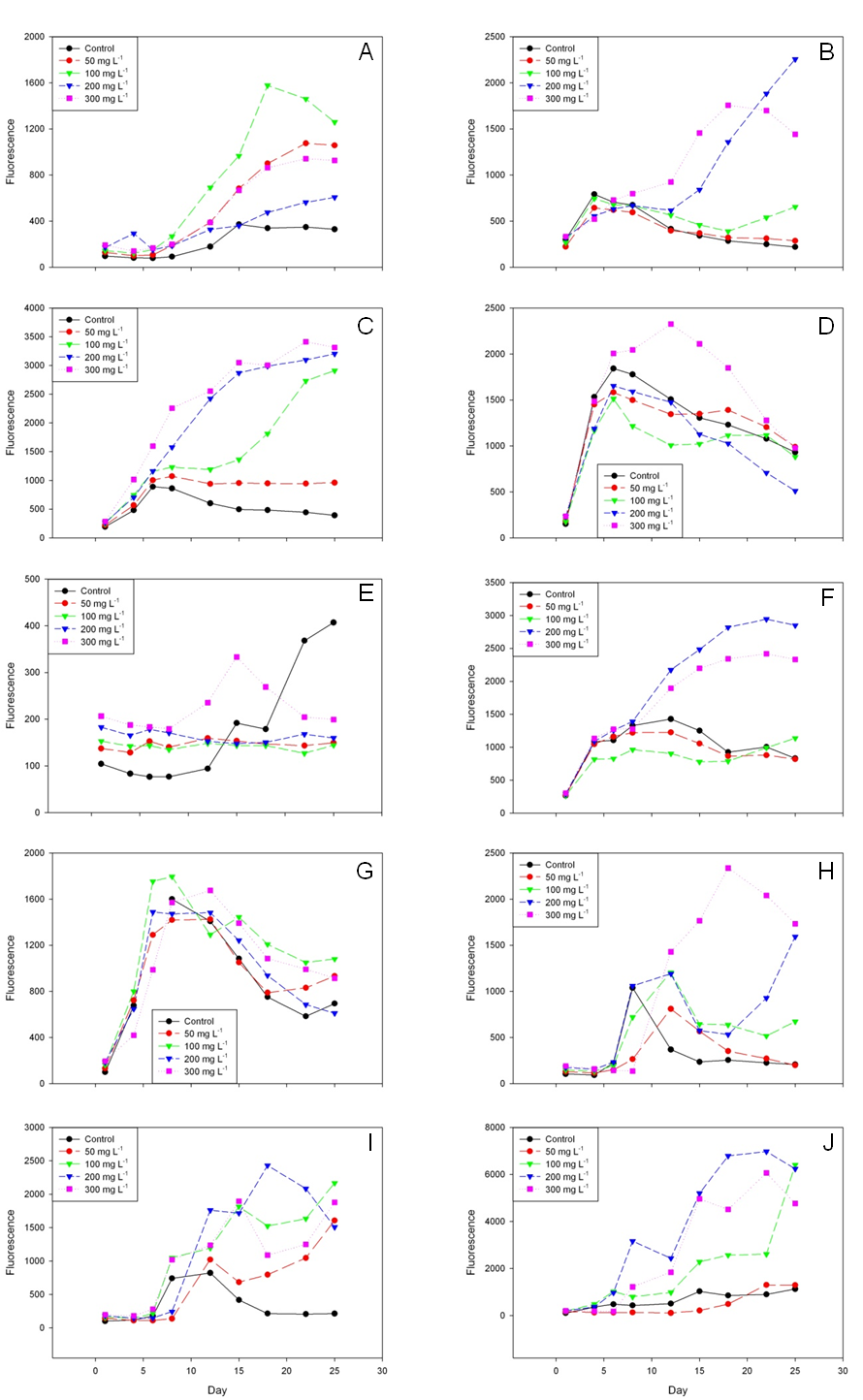

Supplement: Figure S3 — Growth trajectories for each of the ten species used in this experiment. A: Anabaena spp.; B: Navicula subminuscula; C: Scenedesmus quadricauda; D: Nitzschia pusilla; E: Oscillatoria spp.; F: Selanastrum minutum; G: Spirogyra communis; H: Planothidium lanceolatum; I: Tabularia fasciculate; J: Stigeoclonium tenue. (TIF) [file pone.0047130.s003.tif]
