# Supplementary material for: Effects of Nano-Titanium Dioxide on Freshwater Algal Population Dynamics
Source: PLoS One. 2012 Oct 10;7(10):e47130. doi: 10.1371/journal.pone.0047130 (PMC3468504; doi:10.1371/journal.pone.0047130)
Supplement: Table S1 — Characteristics of the soil extract media used in this study, as measured in a companion study. Reprinted with permission from Keller et al., Stability and Aggregation of Metal Oxide Nanoparticles in Natural Aqueous Matrices, Environmental Science and Technology. Copyright 2010, American Chemical Society. (DOCX) [file pone.0047130.s005.docx]

Table S1: **Characteristics of the soil extract media used in this study, as measured in a companion study.** The information provided here is specific to the soil-extract media and titanium dioxide nanoparticles (n-TiO_2_) used in the current study. Reprinted with permission from Keller et al., Stability and Aggregation of Metal Oxide Nanoparticles in Natural Aqueous Matrices, *Environmental Science and Technology*. Copyright 2010, American Chemical Society.

| **Measure (units)** | **Value** |
| --- | --- |
| pH | 8.38 |
| TOC (*μ*M C) | 5,283 |
| UV_254_ (cm^-1^) | 2.872 |
| conductivity (*μ*S) | 372.0 |
| resistivity (mΩ) | 0.003 |
| TDS (mg L^-1^) | 247.00 |
| SO_4_^2-^ (mg L^-1^) | 56.70 |
| Cl- (mg L^-1^) | 103.3 |
| NO_3_^-^ (mg L^-1^) | 2.10 |
| NO_2_^-^ (mg L^-1^) | 0.08 |
| HCO_3_^-^ (mg L^-1^ CaCO_3_) | 61.0 |
| PO_4_^3-^ (mg L^-1^) | 15.00 |
| K^+^ (mg L^-1^) | 22.28 |
| Na^+^ (mg L^-1^) | 55.81 |
| Ca^2+^ (mg L^-1^) | 17.66 |
| Mg^2+^ (mg L^-1^) | 11.37 |
| ionic strength (eq. L^-1^) | 7.18×10^-3^ |
| Al (mg L^-1^) | 2.82 |
| As (mg L^-1^) | 0.00 |
| B (mg L^-1^) | 0.55 |
| Ba (mg L^-1^) | 0.11 |
| Fe (mg L^-1^) | 0.96 |
| I (mg L^-1^) | 2.84 |
| Mn (mg L^-1^) | 0.01 |
| P (mg L^-1^) | 4.85 |
| S (mg L^-1^) | 21.78 |
| Si (mg L^-1^) | 11.87 |
| Sr (mg L^-1^) | 0.10 |
| Ti (mg L^-1^) | 0.01 |
| Zn (mg L^-1^) | 0.01 |
